# Intimate partner violence against women in western Ethiopia: prevalence, patterns, and associated factors

**DOI:** 10.1186/1471-2458-11-913

**Published:** 2011-12-09

**Authors:** Sileshi G Abeya, Mesganaw F Afework, Alemayehu W Yalew

**Affiliations:** 1 Departments of Reproductive Health, Population and Nutrition, School of Public Health, Addis Ababa University, P.O. Box 9086 Addis Ababa, Ethiopia; 2 Departments of Epidemiology and Biostatistics, School of Public Health, Addis Ababa University, Addis Ababa, Ethiopia

**Keywords:** Intimate partner violence, Women, Prevalence, Patterns, Factors

## Abstract

**Background:**

Intimate partner violence against women is the psychological, physical, and sexual abuse directed to spouses. Globally it is the most pervasive yet underestimated human rights violation. This study was aimed at investigating the prevalence, patterns and associated factors of intimate partner violence against women in Western Ethiopia.

**Methods:**

A cross-sectional, population based household survey was conducted from January to April, 2011 using standard WHO multi-country study questionnaire. A sample of 1540 ever married/cohabited women aged 15-49 years was randomly selected from urban and rural settings of East Wollega Zone, Western Ethiopia. Data were principally analyzed using logistic regression.

**Results:**

Lifetime and past 12 months prevalence of intimate partner violence against women showed 76.5% (95% CI: 74.4-78.6%) and 72.5% (95% CI: 70.3-74.7%), respectively. The overlap of psychological, physical, and sexual violence was 56.9%. The patterns of the three forms of violence are similar across the time periods. Rural residents (AOR 0.58, 95% CI 0.34-0.98), literates (AOR 0.65, 95% CI 0.48-0.88), female headed households **(**AOR 0.46, 95% CI 0.27-0.76) were at decreased likelihood to have lifetime intimate partner violence. Yet, older women were nearly four times (AOR 3.36, 95% CI 1.27-8.89) more likely to report the incident. On the other hand, abduction (AOR 3.71, 95% CI 1.01-13.63), polygamy (AOR 3.79, 95% CI 1.64-0.73), spousal alcoholic consumption (AOR 1.98, 95% CI 1.21-3.22), spousal hostility (AOR 3.96, 95% CI 2.52-6.20), and previous witnesses of parental violence (AOR 2.00, 95% CI 1.54-2.56) were factors associated with an increased likelihood of lifetime intimate partner violence against women.

**Conclusion:**

In their lifetime, three out of four women experienced at least one incident of intimate partner violence. This needs an urgent attention at all levels of societal hierarchy including policymakers, stakeholders and professionals to alleviate the situation.

## Background

Violence against women (VAW) is "...any act of gender-based violence that results in, or is likely to result in, physical, sexual or psychological harm or suffering to women, including threats of such acts, coercion or arbitrary deprivation of liberty, whether occurring in public or in private life" [1]. Since women are disproportionately affected than men (95% Vs 5%), gender based violence is often used interchangeably with violence against women [2,3]. Furthermore, the most common and universally occurring (85%) form of VAW is that perpetrated by a husband or other intimate partners [3-5].

Intimate partner violence against women (IPVAW) is the most pervasive yet under estimated social and health problem that occur in pandemic proportions [2-8]. The proportion is comparable to those for cancer, cardiovascular disease, HIV/AIDS, malaria, and traffic accident in the world [2,5,9,10]. In fact, it becomes increasingly known as a health and human rights concern, and prevents women's enjoyment of their fundamental right and freedom that can hinder development [3,4,11].

In spite of the definitions and methodological differences, several population-based studies from around the world indicated that 10%-71% of married or cohabited women have experienced IPVAW [2,5,12,13]. On the other hand, World Health Organization (WHO) multi-country study on VAW in 10 different countries confirmed that the lifetime and current (past 12 months) prevalence of physical or sexual violence ranges between 15 and 71% and 4-54%, respectively. According to the findings of the study, the lowest rates have been found in Japan and the highest in Ethiopia, Peru, and Bangladesh [14].

The root causes of intimate partner violence against women are diverse and there is no single factor that explains further why some individuals are violent, or why violence is more prevalent in some communities than in others [2,4-6]. Rather, several complex and inter connected social and cultural factors are involved. Indeed, all of them are manifestations of unequal power relations between men and women [15]. Moreover, an ecological model for understanding the factors of IPVAW was described at the levels of individual, relationships, community, and society [5,15].

In Ethiopian context, although women represent 49.8% of the population and highly contribute to socio-economic development, they occupy lower status than men. They experience longer working days, low levels of education, and lack of adequate assignments in leadership and decision making positions [16]. However, studies from Ethiopia on IPVAW are few irrespective of different lifestyles, customs and culture of the people [16]. According to a handful of available population based studies from the northern and southern part of the country, the prevalence of IPVAW varies from 50 to 71% during lifetime and 30-54% for past 12 months [17-21].

Yet, in western part of the country where the culture of the community is fairly different, population based study on IPVAW is hardly found. Thus, this research was aimed at investigating the extent, patterns, and associated factors of IPVAW in a sample of women aged 15-49 years living in urban and rural settings of East Wollega Zone, Western Ethiopia.

## Methods

The study was conducted in one urban local government (Nekemte) and rural areas of four districts in East Wollega Zone, which is one of the 18 zones of Oromiya regional state, Ethiopia. East Wollega Zone is located at the western part of the country 331 KMs from Addis Ababa, Ethiopia. In the year 2011, the total population of the zone is 1,340,581 [22]. Oromo is the predominant ethnic group in the zone and *Afan Oromo *is used as a working language [22].

### Study design and population

A cross-sectional population based household survey was carried out between January and April, 2011. As the source population, ever married/cohabited women aged 15-49 years who were residents of the study community for at least 6 months were used. The aforementioned group was selected as it is at the highest risk of intimate partner violence [14].

Adequate sample size was computed using single proportion sample size calculation formula with the inputs of 95% confidence level, 4% margin of error, and 25% non-response rate [23]. Accordingly, a sample size of 1533 women was calculated. However, to represent the urban and rural distribution, 15% of the population from urban and 85% from rural, the sample size was increased to 1600 [21,22].

Respondents were selected principally using multistage sampling technique. Initially, two from six sub-cities found in Nekemte urban local government and eight *kebeles (the lowest administrative unit in the government structure) *from four districts at a distance of 20-30 KMs away from the urban to represent the rural community were randomly selected from 50 *kebeles*. Household census and numbering was done in the selected sub-cities and *kebeles *to fix a sampling frame. After identifying households with the target groups, proportion to sample size allocations were carried out based on the total number of the selected households they have. Ultimately, systematic random sampling was employed to identify respondents from the selected households as a study unit. In a situation when the household has two or more eligible subjects only one was selected by Kish grid (lottery) method to control the potential intra-household correlation [24].

### Data collection

Data was collected by 25 high school completed female interviewers using WHO multi-country study of VAW questionnaire [25]. The questionnaire has been translated to local language (*Afan Oromo*) by experts in both languages and back translated to English by another person to ensure consistency and accuracy. The data collection process was closely supervised by five Health Officers and principal investigators.

The research team was recruited based on qualification, previous experience in data collection and fluency in local language. Moreover, training was given for seven consecutive days in sampling, interview technique, and ethical issues, emphasizing the importance of safety of the participants and interviewers, minimization of under-reporting and maintaining confidentiality. A pre-test study was conducted in one *kebeles *on 10% of the total sample size to practically acquaint participants with the administration of interview process.

A standard field work manual developed by WHO for violence study was adopted and used by the research teams [2]. To ensure the quality of the data and minimize inter-interviewer variation, about 5% of the respondents were re-interviewed at random by principal researchers and supervisors. For that matter few minor differences were detected in the responses given during the second interview.

### Measurements

Variables that have been theoretically, empirically and conceptually linked to IPVAW such as area of residence, age, level of education, occupation, socio-economic status of the household, marital status, alcohol consumption, and husbands fighting habit with another people in the community were taken as independent variables. These and other related variables were categorized into groups where some of them were further sub-divided for bivariate and multivariate analysis.

The dependent variables were considered following conventional definitions of the lifetime and current (past 12 months) experiences of IPVAW. Here, a series of questions were included based on a modified version of the revised Conflict Tactics Scale (CTS2) which guarantees high reliability and constructive validity [26].

The scale lists four questions of psychological abuse to measure items such as insulting the woman, belittlement in front of others, teasing on purpose, and threats to hurt her or someone she cared about. The scale also listed about six questions for physical violence ranking according to its likelihood of causing injuries as moderate like slapping/throwing things, pushing/shoving or severe such as hitting, kicking, beating, choking or burning on purpose, and threatening using a weapon [13]. Additionally, the CTS2 included three questions on sexual violence whether the husbands/partners physically forced to have sexual intercourse when the woman did not want to, or had sexual intercourse when she did not want to because she was afraid of what partner might do, and/or forced to do something sexual that she found humiliating or out of their norms.

### Analysis

The pre-coded responses were double entered into Epi DATA version 3.1 and exported into SPSS version 19 for data checking, cleaning, bivariate and multivariate analysis. Socio-economic status was measured by constructing a wealth index using principal component analysis. Each household was assigned a standardized score that vary depending on whether or not the household owned different assets and the scores were ranked in quintiles [27].

The analysis was focused on the lifetime and current (past 12 months) prevalence of psychological, physical and sexual IPVAW and the association of selected potential socio-demographic, cultural and behavioral factors. Binary logistic regression model was used to identify the characteristics that differentiated ever married/cohabited women who experienced intimate partner violence from those who had not. The results were expressed as crude and adjusted odds ratio relative to the reference category at statistical significance of 95% confidence intervals and P-value of < 0.05. The assumptions of logistic regression were checked to be satisfied.

### Ethical considerations

The research was approved for scientific and ethical integrity by institutional review board in the College of Health Sciences, Addis Ababa University. The study strictly followed WHO guideline on ethical issues related to violence research [2,28,29]. All interviews took place in a complete privacy. Verbal consent from all respondents and/or assent from respondents aged 15-17 years were secured. During data collection interviewees with serious psychological distress were referred to Nekemte Hospital for counseling. Information regarding available local services was shared to all respondents.

## Results

### Socio-demographic characteristics

A total of 1540 study subjects were attended the interview making a response rate of 96.3%. The socio-demographic characteristics of the women and their partners were described in table 1. Most of the respondents (84.2%) were residing in rural setting and 78.6% were in the age range of 20-34 years. The mean age of the respondents is 28.4 years (± 5.7SD). The vast majorities (98.7%) of the respondents were ever married at the time of the interview, predominantly Christian (97.5%) and Oromo (96.4%) in their religion and ethnicity.

**Table 1:** Socio demographic characteristics of ever married/cohabited women age 15-49 years and their husbands/partners in East Wollega Zone, Western Ethiopia, January to April, 2011

Characteristics (Variables) n = 1540	Number	Percent
**Age of respondents**		
15-19 years 20-34 years 35- 49 years	28 1,208 304	1.8 78.4 19.8

**Marital status**		
Currently married Currently cohabited Separated/divorced/widowed	1420 20 100	92.2 1.3 6.5

**Education of respondents**		
No formal education Primary (1-6^th ^grade) Secondary and above (≥ 7^th ^grade)	919 399 222	59.7 25.9 14.4

**Current occupation of respondents**		
No job Trade activities Employed into different sectors Female headed Housemaid Others^†^	1283 63 24 74 25 71	83.3 4.0 1.6 4.8 1.6 4.6

**Wealth quintile**		
Poorest Poor Medium Rich Richest	305 320 318 269 328	19.8 20.8 20.6 17.5 21.3

**Current husbands/partners age**		
18-24 years 25-34 years 35-49 years ≥ 50 years	69 669 649 153	4.5 43.4 42.1 9.9

**Partner's education level (n = 1529)**		
No formal education Primary (1-6 grade) Secondary (≥ 7th grade)	521 559 449	34.1 36.6 29.4

**Partner's occupation**		
Employee^§^ Daily labourer, student Petty trader Farmer No job, retired	146 129 110 1039 116	9.5 8.4 7.1 67.5 7.5

Others^† ^include students and unspecified job

Employee^§ ^includes professional, semi skilled, soldiers and police

Nearly about three fifth (59.7%) of the respondents had no formal education. More than four in every five (83.3%) had no job, and 59.5% moved to the study area due to marriage and work related conditions after born and brought up in other localities where immediate parents were residing.

According to the report from the interviewed respondents, the mean age of the current husbands/partners has been 37.1 years (± 14.5 SD). Unlike the respondents, the husbands'/partners' age ranged from 18-88 years. More than one third (34.1%) of husbands/partners had no formal education, and 67.5% engaged into agricultural occupations (Table 1).

The larger proportion (63.1%) of the respondents have got marriage/cohabitation in the age range of 15-19 years, while for 2.3% of them, the marriage/cohabitation was below the age of 15 years. Accordingly, the mean age of first marriage/cohabitation for the women was 18.6 years (± 2.6 SD). On the other hand, 40 (2.6%) of them were divorced when the study was conducted out of which, 17 (42.2%) of the divorce was decided by husbands/partners, 12 (30%) by the respondents and 10 (25%) was initiated by both partners.

For about a quarter (26.3%) of the couple the initiation of marriage was not based on their own choices. In similar manner, nearly about one in three (30.1%) of them have never conducted marriage ceremony when they started to live together. Besides, not surprisingly 7.2% of the women were reported in having had marriage by abduction. On the other hand, more than one in ten (12.1%), two in three (64.8%), and more than two in three (68.4%) of the respondents had married to polygamous, alcohol drunker, and hostile husbands/partners, in that order (Table 2).

**Table 2:** Cultural and behavioral characteristics of ever married/cohabited women aged 15-49 years and their partners in East Wollega Zone, Western Ethiopia, January to April, 2011

Variables	Number	Percent
**Age at first marriage/cohabiting (n = 1540)**		
10-14 years 15-19 years 20-24 years ≥ 25 years	36 972 452 58	2.3 63.1 29.4 3.8

**Mean age at first marriage/cohabited in years (n = 1540)**	18.6 ± 2.6SD	

**Initiation of marriage/cohabitation (n = 1540)**		
Both (either woman or man) choose Family and others choose Abduction	1136 292 112	73.8 19.0 7.3

**Marriage ceremony (n = 1540)**		
None Civil marriage Religious marriage Customary marriage	463 63 390 624	30.1 4.1 25.3 40.5

**Initiation of divorce (n = 40)**		
Respondent Husband/partner Both (respondent and partner) Family	12 17 10 1	30.0 42.5 25.0 2.5

**Number of children (n = 1540)**		
0 1-2 ≥ 3	70 626 844	4.5 40.6 54.8

**Respondent drink alcohol (n = 1539)**		
Never Light (occasional) Heavy (frequently)	681 554 304	44.2 36.0 19.8

**Situation of marriage for current husbands/partners (n = 1540)**		
Monogamous Polygamous Refused to answer	1351 186 3	87.7 12.1 0.2

**Partner's drink alcohol (n = 1512)**		
Never Light (occasional) Heavy (frequently)	532 493 487	35.2 32.6 32.2

**Husbands/partners fighting habit (n = 1503)**		
No Yes	449 1054	29.2 68.4

### Prevalence and forms of violence

The occurrences and patterns, timing and frequencies of different forms of IPVAW (psychological, physical, and sexual) were assessed. This is done as the lifetime and current prevalence are useful in reporting the time periods, as recall bias ought to be less in studies of such serious life threatening experiences than inquiring about less sensitive matters [30,31].

### Psychological violence

About two third (66.9%) of the participating women were verbally insulted and made feel bad about themselves for at least once in their lifetime. One for every three (34.8%) women was ever humiliated in front of other persons. Moreover, in their lifetime 38.9% were intimidated, and 18.3% frightened someone they cared about. In similar manner, for 59.4%, 31.5%, 6.5%, and 15.5% of the respondents, these were happened for at least once during the past 12 months, correspondingly. Generally, the prevalence of psychological violence was 70.2%, 95% CI 67.9-72.5% during lifetime and 63.9%, 95% CI 61.5-66.3% in current experiences (Table 3).

**Table 3:** Life time and past 12 months prevalence and frequency of different forms of IPVAW among ever married/cohabited women age 15-49 years in East Wollega Zone, Western Ethiopia, January to April, 2011

Forms of IPVAW (N = 1540)	Lifetime	Past 12 months	Frequency in the current (past 12 months)			Frequency before 12 months		
	
	Number %	Number %	Once	Few times '(2-5)	Many times (> 5)	Once	Few times (2-5)	Many times (> 5)
**Psychological/Emotional violence**								
• Insulted/made feel bad	1,030 (66.9)	914 (59.4)	230 (24.9)	565 (36.7)	119 (7.7)	207 (13.4)	539 (35.0)	278 (18.1)
• Humiliated in front of others	536 (34.8)	485 (31.5)	190 (12.3)	219 (14.2)	76 (4.9)	113 (7.3)	303 (19.7)	120 (7.8)
• Intimidated on purpose	599 (38.9)	524 (6.5)	190 (12.3)	271 (17.6)	64 (4.2)	134 (8.7)	327 (21.2)	131 (8.5)
• Threaten/hurt/frighten someone they care about	281 (18.3)	242 (15.7)	114 (7.4)	96 (6.2)	32 (2.1)	79 (5.1)	143 (9.3)	56 (3.6)
❖ **At least one episode of psychological abuse**	**1081 (70.2)**	**984 (63.9)**	**394 (25.6)**	**680 (44.2)**	**207 (13.4)**	**300 (19.5)**	**746 (48.4)**	**366 (23.8)**

**Physical violence**								
**Moderate physical violence**	**961 (62.4)**	**961 (62.3)**	**288 (18.7)**	**595 (38.6)**	**118 (7.7)**	**237 (15.4)**	**631 (41.0)**	**262 (17.0)**
• Slapped/Thrown some thing	893 (58.0)	797 (51.6)	222 (14.4)	495 (32.1)	78 (5.1)	179 (11.6)	511(33.2)	200 (13.0)
• Pushed or shoved	621 (40.3)	540 (35.1)	159 (10.3)	324 21.0)	59 (3.8)	145 (9.4)	371(24.1)	108 (7.0)
**Sever physical violence**	**835 (54.2)**	**757 (49.2)**	**230 (14.9)**	**521 (33.8)**	**82 (5.3)**	**161 (10.5)**	**517 (33.6)**	**266 (17.3)**
• Hit with fist or something else	649 (42.1)	581 (37.7)	164 (10.6)	375 (24.4)	42 (2.7)	110 (7.1)	367(23.8)	170 (11.0)
• Kicked, dragged or beat	570 (37.0)	490 (31.8)	97(6.3)	338 (21.9)	57(3.7)	66 (4.3)	305(19.8)	193 (12.5)
• Choked or burnt	142 (9.2)	125 (8.1)	39 (2.5)	76 (4.9)	9 (0.6)	21(1.4)	103(6.7)	23 (1.5)
• Threatened or used weapon (gun, knife)	86 (5.6)	80 (5.2)	27 (1.8)	47 (3.1)	7 (0.5)	16 (1.0)	55(3.6)	23(1.5)
❖ **At least one episode of physical violence**	**1056 (68.6)**	**964 (62.6)**	**379 (24.6)**	**749 (48.6)**	**160 (10.4)**	**290 (18.8)**	**769 (49.9)**	**388 (25.2)**

**Sexual violence**								
• Physically forced to have sex	904 (58.7)	786 (51.0)	160 (10.4)	417(27.1)	208(13.5)	126 (8.2)	432 (28.1)	333 (21.6)
• Having sex because of fear of partner	712 (46.2)	622 (40.4)	116 (7.5)	317 (20.6)	199 (12.9)	105 (6.8)	319 (20.7)	282 (18.3)
• Sex that is degrading/humiliating	127(8.3)	108 (7.0)	29 (1.9)	70 (4.5)	10 (0.6)	26 (1.7)	79 (5.1)	24 (1.6)
❖ **At least one episode of sexual violence**	**948 (61.6)**	**847 (55.0)**	**204 (13.2)**	**500 (32.5)**	**250 (16.2)**	**165 (10.7)**	**499 (32.4)**	**394 (25.6)**

**At least one of the three violence**	**1178 (76.5)**	**1117 (72.5)**						

NB- Percentage in each column may not add 100 as respondents can report more than one

### Physical violence

Sixty two percent of the respondents ever experienced being slapped and shoved by their husbands/partners across their lifetime. These are the most common acts of moderate physical violence. Most women were reported to have beaten up, punched, dragged and knocked- which are acts of severe physical violence. Other severe acts of physical violence including burning and chocking were also common. In this case, the proportion of women who had experiences of severe physical violence was 54.2% in lifetime and 49.2% in past 12 months. This shows that more than three quarter (79% not shown) who experienced any physical violence had severe physical aggression in lifetime. Besides, for all cases the violence was exerted as repeated acts as described in table 3. Generally, 1056 (68.6%, 95% CI 66.3-70.9%) of the women experienced at least one or more incidents of physical violence in their lifetime, and for 964 (62.6%, 95 CI 60.2-65.0%) the incidents were happened during the past 12 months.

### Sexual violence

About 59% of the respondents reported that at some point in their life time, their husbands/partners had forced them to have sexual intercourse without their interest or consent and in 51% of them it was happened in the preceding 12 months of the survey. In addition, 46.2% and 40.4% of respondents experienced sexual intercourse during their lifetime and current relationship due to fear of their husbands/partners. The proportion of women who had been forced into a humiliating sexual acts like pornographic show or practice of sexual acts out of their norms were 8.3% and 7.0% during lifetime and past 12 months, respectively. Overall, 948 (61.6%, 95% CI 59.2-64.0%) and 847 (55.0%, 95% CI 52.5-57.5%) of the women have reported to have at least one incident of sexual violence in their lifetime and past 12 months, respectively.

### Prevalence and patterns of IPVAW-summary measures

In this study, 1178 (76.5%, 95% CI 74.4-78.6%) of the respondents experienced IPVAW in one form or another at some point in their lifetime. Besides, 1117 (72.5%, 95% CI 70.3-74.7%) of them experienced the same incidents in the past 12 months. Most acts of IPVAW were part and a pattern of continuing abuse rather than being an isolated event (for detail, Table 3).

Although it is expected that the overall prevalence of IPVAW in the past 12 months would be substantially lower than the women's lifetime experiences, much smaller variation with similar patterns were observed (Table 3). Likewise, most respondents experienced each act of IPVAW from once to many times in lifetime and past 12 months.

The proportion of respondents experienced the three forms of IPVAW during current (past 12 months), previous (before one year), and lifetime marital or cohabiting relationship indicated that out of 76.5% who had gone through lifetime IPVAW, about 75.8% had the incident in the previous and 72.5% in the past 12 months. Similarly, the patterns were 70.2%, 70.0% and 63.9% for psychological violence, 68.6%, 68.6% and 62.2% for physical, and 61.6%, 61.0% and 55.0% for sexual violence. These patterns revealed, the percentage of current, previous, and lifetime experiences of IPVAW are almost similar.

**Figure 1:**
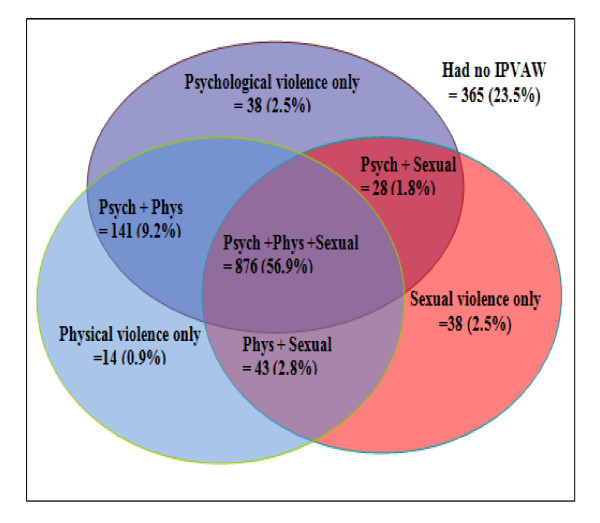
Venn diagram illustrating overlaps between lifetime experiences of psychological, physical and sexual violence reported by ever married/cohabited women aged 15-49 years in East Wollega Zone, Western Ethiopia, January to April, 2011

The patterns for the joint occurrences of different forms of IPVAW are shown in figure 1. Hence, physical violence (0.9%) is less likely to occur in isolated form when compared to psychological and sexual violence which accounts 2.5% for each of the isolated occurrence. However, the joint occurrences of psychological and physical violence account 9.2%. It exceeds the other two overlapping patterns of 1.8% and 2.8% for psychological + sexual violence and of physical + sexual violence, respectively. On the other hand, the greater proportion (56.9%) of women experienced multiple forms of violence from their intimate partners at the same time.

### Factors associated with IPVAW

In the final model, a number of socio-demographic factors were identified as significant predictors of lifetime and current experiences of IPVAW (Table 4). Compared to urban dwellers, rural dwellers were less likely to report lifetime IPVAW (AOR 0.58, 95% CI 0.34-0.98). However, this association was not significant after controlling for other factors in past 12 months. In the same way, compared to respondents aged 15-19 years, those from 35-49 years were about four times (AOR 3.36, 95% CI 1.27-8.89) and three times (AOR 2.75, 95% CI 1.10-6.86) more likely to report lifetime and current IPVAW.

**Table 4:** Odds Ratios predicting IPVAW among ever married/cohabited women aged 15-49 by selected socio-demographic variables in East Wollega Zone, Western Ethiopia, January to April, 2011

Variables	Lifetime IPVAW			Recent (past 12 months) IPVAW		
	
	No (%)	COR (95%CI)	AOR (95% CI)	No (%)	COR (95%CI)	AOR (95% CI)
**Residence**						
Urban	214 (87.7)	1.00	1.00	17 (80.3)	1.00	1.00
Rural	965 (74.4)	**0.41 (0.27 **to 0.61)**	**0.58 (0.34 **to 0.98)*	33 (71.1)	**0.60 (0.43 **to 0.84)*	0.92 (0.58 to 1.47)

**Age of respondent**						
15-19 years	18 (64.3)	1.00	1.00	18 (64.3)	1.00	1.00
20-34 years	901 (74.5)	1.62 (0.74 to 3.56)	1.56 (0.63 to 3.85)	850 (70.4)	**1.35 (1.02 **to 1.78)*	1.25 (0.54 to 2.91)
35- 49 years	260 (85.5)	**3.28 (1.42 **to 7.58)*	**3.36 (1.27 **to 8.89)*	249 (81.9)	**1.38 (1.02 **to 1.86)*	**2.75 (1.10 **to 6.86)*

**Relationship with current partner**						
Currently married	1079 (76.0)	1.00	1.00	1026 (72.3)	1.00	----
Currently cohabited	14 (70.0)	0.74 (0.28 to 1.93)	0.81 (0.27 to 2.44)	12 (60.0)	0.58 (0.23 to 1.42)	
Divorced, separated, widowed	85 (85.0)	**1.79 (1.02 **to 3.14)*	1.64 (0.87 to 3.10)	79 (79.0)	1.45 (0.88 to 2.37)	

**Education level of respondents**						
No formal education	710 (77.3)	1.00	1.00	22 (74.3)	1.00	1.00
Primary (1-6 grade)	289 (72.4)	0.82 (0.57 to 1.18)	**0.65 (0.48 **to 0.88)*	16 (67.2)	**0.71 (0.55 **to 0.91)*	**0.63 (0.48 **to 0.84)*
Secondary and above (≥ 7)	180 (80.6)	**0.63 (0.42 **to 0.94)*	0.72 (0.45 to 1.17)	22 (74.8)	1.02 (0.73 to 1.44)	0.71 (0.46 to 1.10)

**Occupation of respondents**						
No job	1020 (76.8)	1.00	1.00	963 (72.5)	1.00	1.00
Student/employee/trader	90 (79.6)	1.18 (0.74 to 1.90)	0.87 (0.50 to 1.52) 5.29 (0.66 to 42.69)	87 (77.0)	1.27 (0.81 to 2.00)	1.18 (0.71 to 1.97)
Housemaid	24 (96.0)	7.25 (0.98 to 53.79)	**0.46 (0.27 **to 0.76)*	23 (92.0)	**4.36 (1.02 **to 18.58)*	3.99 (0.87 to 18.37)
Farmers (female headed)	44 (59.5)	**0.44 (0.27 **to 0.72)*		44 (59.5)	**0.56 (0.34 **to 0.90)*	0.58 (0.35 to 0.97)

**Partner's education level**						
No formal education	413 (79.3)	1.00	1.00	394 (75.6)	1.00	1.00
Primary (1-6 grade)	411 (73.5)	**0.73(0.55 **to 0.96)*	**0.73(0.55 **to 0.99)*	393 (70.3)	**0.76 (0.58 **to 1.00)*	0.73 (0.59 to 1.04)
Secondary and above (≥ 7)	344 (76.6)	0.86 (0.63 to 1.16)	0.74 (0.43 to 1.29)	320 (71.3)	0.80 (0.60 to 1.04)	**0.71(0.51 **to 0.98)*

**Difference of education between partners**						
Women higher	110 (71.7)	1.00	1.00	100 65.8)	1.00	1.00
Equal educational status	496 (79.7)	**1.55 (1.04 **to 2.33)*	1.63 (0.97 to 2.73)	470 (75.6)	**1.61 (1.20 **to 2.36)*	**1.67 (1.05 **to 2.68)*
Woman's lower	563 (74.6)	1.16 (0.78 to 1.71)	1.36 (0.67 to 2.76)	537 (71.1)	1.28 (0.86 to 1.86)	1.44 (0.76 to 2.76)

**Age of current husbands/partners**						
18-29 years	250 (71.5)	1.00	1.00	250 (67.8)	1.00	1.00
30-39 years	532 (77.5)	**1.37 (1.03 **to 1.82)*	1.06 (0.77 to 1.44)	532 (73.9)	**1.35 (1.02 **to 1.77)*	1.12 (0. 84 to 1.51)
≥ 40 years	335 (78.9)	**1.49 (1.08 **to 2.05)*	0.80 (0.53 to 1.17)	335 (74.3)	**1.38 (1.02 **to 1.86)*	0.84 (0.59 to 1.20)

**Partners occupation**						----
Employee†	120 (82.2)	1.00	1.00	112 (76.7)	1.00	
Daily labourer, student	104 (80.6)	0.90 (0.49 to 1.66)	0.88 (0.46 to 1.68)	98 (76.0)	0.96 (0.55 to 1.68)	
Petty trader	91 (82.7)	1.04 (0.54 to 1.99)	1.41 (0.50 to 2.83)	82 (74.5)	0.89 (0.50 to 1.58)	
Farmer	767 (73.7)	**0.61 (0.39 **to 0.95)*	0.75 (0.43 to 1.29)	734 (70.6)	0.73 (0.49 to 1.10)	
No job, retired	97 (83.6)	1.11 (0.58 to 2.12)	1.11 (0.56 to 2.18)	91 (78.4)	1.12 (0.62 to 1.99)	

**Wealth quintile**						
Poorest	250 (82.0)	1.00	1.00	239 (78.4)	1.00	1.00
Poor	239 (74.7)	**0.65 (0.44 **to 0.96)*	**0.64 (0.43 **to 0.96)*	228 (71.3)	**0.68 (0.48 **to 0.99)*	0.70 (0.48 to 1.02)
Medium	241 (75.8)	0.69 (0.47 to 1.02)	0.71 (0.47 to 1.07)	228 (71.7)	0.70 (0.49 to 1.01)	0.76 (0.52 to 1.11)
Richer	197 (73.2)	**0.60 (0.40 **to 0.90)*	**0.61 (0.40 **to 0.94)*	186 (69.1)	**0.62 (0.43 **to 0.90)*	**0.65 (0.44 **to **0.97**)*
Richest	251 (76.5)	0.72 (0.49 to 1.06)	**0.66(0.44 **to 0.99)*	236 (72.0)	0.71 (0.49 to 1.02)	0.69 (0.47 to.02)

**Variables**	**Lifetime IPVAW**			**Recent (past 12 months) IPVAW**		
	**No (%)**	**COR (95%CI)**	**AOR (95% CI)**	**No (%)**	**COR (95%CI)**	**AOR (95% CI)**

**Residence**						
Urban	214 (87.7)	1.00	1.00	17 (80.3)	1.00	1.00
Rural	965 (74.4)	**0.41 (0.27 **to 0.61)**	**0.58 (0.34 **to 0.98)*	33 (71.1)	**0.60 (0.43 **to 0.84)*	0.92 (0.58 to 1.47)

**Age of respondent**						
15-19 years	18 (64.3)	1.00	1.00	18 (64.3)	1.00	1.00
20-34 years	901 (74.5)	1.62 (0.74 to 3.56)	1.56 (0.63 to 3.85)	850 (70.4)	**1.35 (1.02 **to 1.78)*	1.25 (0.54 to 2.91)
35- 49 years	260 (85.5)	**3.28 (1.42 **to 7.58)*	**3.36 (1.27 **to 8.89)*	249 (81.9)	**1.38 (1.02 **to 1.86)*	**2.75 (1.10 **to 6.86)*

**Relationship with current partner**						
Currently married	1079 (76.0)	1.00	1.00	1026 (72.3)	1.00	----
Currently cohabited	14 (70.0)	0.74 (0.28 to 1.93)	0.81 (0.27 to 2.44)	12 (60.0)	0.58 (0.23 to 1.42)	
Divorced, separated, widowed	85 (85.0)	**1.79 (1.02 **to 3.14)*	1.64 (0.87 to 3.10)	79 (79.0)	1.45 (0.88 to 2.37)	

**Education level of respondents**						
No formal education	710 (77.3)	1.00	1.00	22 (74.3)	1.00	1.00
Primary (1-6 grade)	289 (72.4)	0.82 (0.57 to 1.18)	**0.65 (0.48 **to 0.88)*	16 (67.2)	**0.71 (0.55 **to 0.91)*	**0.63 (0.48 **to 0.84)*
Secondary and above (≥ 7)	180 (80.6)	**0.63 (0.42 **to 0.94)*	0.72 (0.45 to 1.17)	22 (74.8)	1.02 (0.73 to 1.44)	0.71 (0.46 to 1.10)

**Occupation of respondents**						
No job	1020 (76.8)	1.00	1.00	963 (72.5)	1.00	1.00
Student/employee/trader	90 (79.6)	1.18 (0.74 to 1.90)	0.87 (0.50 to 1.52) 5.29 (0.66 to 42.69)	87 (77.0)	1.27 (0.81 to 2.00)	1.18 (0.71 to 1.97)
Housemaid	24 (96.0)	7.25 (0.98 to 53.79)	**0.46 (0.27 **to 0.76)*	23 (92.0)	**4.36 (1.02 **to 18.58)*	3.99 (0.87 to 18.37)
Farmers (female headed)	44 (59.5)	**0.44 (0.27 **to 0.72)*		44 (59.5)	**0.56 (0.34 **to 0.90)*	0.58 (0.35 to 0.97)

**Partner's education level**						
No formal education	413 (79.3)	1.00	1.00	394 (75.6)	1.00	1.00
Primary (1-6 grade)	411 (73.5)	**0.73(0.55 **to 0.96)*	**0.73(0.55 **to 0.99)*	393 (70.3)	**0.76 (0.58 **to 1.00)*	0.73 (0.59 to 1.04)
Secondary and above (≥ 7)	344 (76.6)	0.86 (0.63 to 1.16)	0.74 (0.43 to 1.29)	320 (71.3)	0.80 (0.60 to 1.04)	**0.71(0.51 **to 0.98)*

**Difference of education between partners**						
Women higher	110 (71.7)	1.00	1.00	100 65.8)	1.00	1.00
Equal educational status	496 (79.7)	**1.55 (1.04 **to 2.33)*	1.63 (0.97 to 2.73)	470 (75.6)	**1.61 (1.20 **to 2.36)*	**1.67 (1.05 **to 2.68)*
Woman's lower	563 (74.6)	1.16 (0.78 to 1.71)	1.36 (0.67 to 2.76)	537 (71.1)	1.28 (0.86 to 1.86)	1.44 (0.76 to 2.76)

**Age of current husbands/partners**						
18-29 years	250 (71.5)	1.00	1.00	250 (67.8)	1.00	1.00
30-39 years	532 (77.5)	**1.37 (1.03 **to 1.82)*	1.06 (0.77 to 1.44)	532 (73.9)	**1.35 (1.02 **to 1.77)*	1.12 (0. 84 to 1.51)
≥ 40 years	335 (78.9)	**1.49 (1.08 **to 2.05)*	0.80 (0.53 to 1.17)	335 (74.3)	**1.38 (1.02 **to 1.86)*	0.84 (0.59 to 1.20)

**Partners occupation**						----
Employee†	120 (82.2)	1.00	1.00	112 (76.7)	1.00	
Daily labourer, student	104 (80.6)	0.90 (0.49 to 1.66)	0.88 (0.46 to 1.68)	98 (76.0)	0.96 (0.55 to 1.68)	
Petty trader	91 (82.7)	1.04 (0.54 to 1.99)	1.41 (0.50 to 2.83)	82 (74.5)	0.89 (0.50 to 1.58)	
Farmer	767 (73.7)	**0.61 (0.39 **to 0.95)*	0.75 (0.43 to 1.29)	734 (70.6)	0.73 (0.49 to 1.10)	
No job, retired	97 (83.6)	1.11 (0.58 to 2.12)	1.11 (0.56 to 2.18)	91 (78.4)	1.12 (0.62 to 1.99)	

**Wealth quintile**						
Poorest	250 (82.0)	1.00	1.00	239 (78.4)	1.00	1.00
Poor	239 (74.7)	**0.65 (0.44 **to 0.96)*	**0.64 (0.43 **to 0.96)*	228 (71.3)	**0.68 (0.48 **to 0.99)*	0.70 (0.48 to 1.02)
Medium	241 (75.8)	0.69 (0.47 to 1.02)	0.71 (0.47 to 1.07)	228 (71.7)	0.70 (0.49 to 1.01)	0.76 (0.52 to 1.11)
Richer	197 (73.2)	**0.60 (0.40 **to 0.90)*	**0.61 (0.40 **to 0.94)*	186 (69.1)	**0.62 (0.43 **to 0.90)*	**0.65 (0.44 **to **0.97**)*
Richest	251 (76.5)	0.72 (0.49 to 1.06)	**0.66(0.44 **to 0.99)*	236 (72.0)	0.71 (0.49 to 1.02)	0.69 (0.47 to.02)

Adjusted for all variables in the model

COR - Crude odds Ratio, AOR - Adjusted Odds Ratio, CI: confidence interval.* P- Value < 0.05, ** *P*- Value < 0.001, †- Professional, semi skilled, soldiers, police

Note: - variables not entered in the model because they were not found significant in bivariate analysis

On the other hand, the protective effects of education for both women as victim and men as perpetrator were found to be significant in the lifetime and current experiences of IPVAW after controlling for age, occupation and socio-economic statuses. However, those women who have equal educational statuses with their husbands/partners were (AOR 1.67, 95% CI 1.05-2.68) more likely to report current experiences of IPVAW than women with greater educational status of their husbands/partners. This association was also noteworthy in lifetime experiences of IPVAW before adjusting for the variables in the model. Similarly, female headed respondents engaged into different working condition were (AOR 0.46, 95% CI 0.27-0.76) less likely to report lifetime IPVAW compared to jobless women.

Compared to respondents from the poorest household, those respondents from poor (AOR 0.65, 95% CI 0.44-0.97), the richer (AOR 0.61, 95% CI 0.40-0.94), and the richest households were (AOR 0.66, 95% CI 0.44-0.99) less likely to report lifetime IPVAW. Similarly, these associations were found significant for current experiences of IPVAW (Table 4).

Table 5 presents the association of cultural and behavioral factors with IPVAW. It shows that women married/cohabited by abduction (AOR 3.71, 95% CI 1.01-13.63), to polygamous partners (AOR 3.79, 95% CI 1.64-8.73), to heavy alcohol drunkard (AOR 1.98, 95% CI 1.21-3.22), and to hostile partners (AOR 3.96, 95% CI 2.52-6.20) remained associated with increased experiences of lifetime IPVAW. These were also found significant in the past 12 months experiences of IPVAW.

**Table 5:** Odds Ratios predicting IPVAW among ever married/cohabited women aged 15-49 by selected cultural and behavioral variables in East Wollega Zone, Western Ethiopia, and January to April, 2011

Variables	Lifetime IPVAW			Current (past 12 months) IPVAW		
	
	No (%)	COR (95%CI)	AOR (95% CI)	No (%)	COR (95%CI)	AOR (95% CI)
**Initiation of marriage or choose (n = 1540)**						
Both	1026 (75.5)	1.00	1.00	972 (71.6)	1.00	1.00
Family and others	88 (78.6)	1.19 (0.74 to 1.90)	1.11 (0.56 to 2.19)	82 (73.2)	1.08 (0.70 to 1.67)	0.87 (0.46 to 1.66)
Abduction	63 (91.3)	**3.40 (1.50 **to 7.93)*	**3.71 (1.01 **to 13.63)*	61 (88.4)	**3.02 (1.43 **to 6.37)*	3.02 (0.93 to 9.76)

**Marriage ceremony**						
No	379 (82.1)	1.00	1.00	353 (71.8) 764 (70.2)	1.00	1.00
Yes	800 (74.2)	**0.63 (0.48 **to 0.83)*	0.81 (0.83 to 1.85)		**0.66 (0.51 **to 0.86)*	0.99 (0.61 to 1.61)

**Dowry/bride Price**						
No	233 (82.6)	1.00	1.00	218 (77.3)	1.00	1.00
Yes	942 (75.0)	**0.63 (0.45 **to 0.88)*	0.80 (0.77 to 2.00)	896 (71.5)	**0.74 (0.54 **to 0.99)*	0.96 (0.55 to 1.68)

**Current partners**						
Monogamous	1012 (74.9)	1.00	1.00	960 (71.1) 154 (82.8	1.00	1.00
Polygamous	163 (87.6)	**2.37 (1.51 **to 3.74)**	**3.79 (1.64 **to 8.73)*		**1.96 (1.32 **to 2.92)*	**2.51 (1.26 **to 4.97)**

**Extra marital affairs of husband**						
No	884 (73.1)	1.00	1.00	837 (89.2)	1.00	1.00
Yes	133 (89.3)	**3.06 (2.03 **to 4.62)**	1.34(0.71 to 2.52)	222 (85.1)	**2**.**53 (1.76 **to 3.63)**	1.28 (0.72 to 2.29)

**Respondent drink alcohol**						
Never	530 (77.8)	1.00	1.00	485 (71.2)	1.00	1.00
Light (occasional)	392 (70.6)	**0.68 (0.53 **to 0.88)*	**0.59 (0.41 **to 0.84)*	380 (68.6)	0.88 (0.69 to 1.13)	0.82 (0.54 to 1.25)
Heavy	256 (84.2)	**1.52 (1.06 **to 2.17)*	1.07 (0.68 to 1.67)	251 (82.6)	**1.91(1.36 **to 2.69)*	1.51 (0.87 to 2.60)

**Partner's drink alcohol (n = 1512)**						
Never	392 (73.7)	1.00	1.00	361 (67.9)	1.00	1.00
Light (occasional)	341 (69.2)	0.80 (0.61 to 1.05)	0.86 (0.56 to 1.32)	329 (66.7)	0.95 (0.73 to 1.23)	0.92 (0.60 to 1.41)
Heavy	424 (78.1)	**2.40 (1.73 **to 3.34)**	**1.98 (1.21 **to 3.22)*	408 (83.8)	2.47 (1.81-3.31)*	**1.88 (1.17 **to 3.01)*

**Fighting habit of partner**						
No	714 (70.3) 412 (91.5)	1.00	1.00	689 (65.4)	1.00	1.00
Yes		**4.57 (3.20 **to 6.53)**	**3.96 (2.52 **to 6.20)**	403 (89.8)	4.64 (3.34-6.46)*	**4.17 (2.65 **to 6.55)**

**Age at first marriage**						
10-14 years	23 (63.9)	1.00	1.00	21 (58.3)	1.00	1.00
15-19 years	738 (75.9)	1.78 (0.89 to 3.58)	1.80 (0.78 to 4.15)	707 (72.7)	1.91 (0.97 to 3.75)	**3.41 (1.31 **to 8.89)*
20-24 years	359 (79.4)	**2.18 (1.07 **to 4.47)*	1.96 (0.83 to 4.62)	334 (73.9)	**2.02 (1.01 **to 4.05)*	**2.93 (1.10 **to 7.78)*
≥ 25 years	47 (79.3)	2.17 (0.85 to 5.50)	2.63 (0.87 to 7.99)	44 (75.9)	2.25 (0.92 to 5.49)	**4.26 (1.27 **to 14.2)*

**First sexual intercourse**						
Wanted	817 (73.9)	1.00	1.00	773 (69.9)	1.00	1.00
Coerced†	359 (83.3)	**1.76 (1.32 **to 2.35)**	1.19 (0.77 to 1.86)	342 (79.4)	**1.66 (1.27 **to 2.16)**	1.27 (0.84 to 1.93)

**Witnessed family violence**						
No	325 (68.7)	1.00	1.00	302 (63.8)	1.00	1.00
Yes	764 (81.4)	**2.00 (1.54 **to 2.56)**	**1.65 (1.14 **to 2.38)*	731 (77.8)	**1.99 (1.56 **to 2.54)**	**1.66 (1.17 **to 2.37)*

**Women heard/seen violence as child**						
No	290 (69.0)	1.00	1.00	268 (63.8)	1.00	1.00
Yes	824 (81.0)	**1.91 (1.48 **to 2.48)**	1.49 (1.01 to 2.19)	788 (77.5)	**1.95 (1.52 **to 2.50)**	1.35 (0.98 to 1.86)

**History of violence of mother-in-law**						
No	446 (73.1)	1.00	1.00	415 (68.0)	1.00	1.00
Yes	415 (81.5)	**1.62 (1.22 **to 2.16)*	1.05 (0.60 to 1.68)	398 (78.2)	**1.69 (1.29 **to 2.21)**	1.04 (0.66 to 1.63)

Adjusted for all variables in the model

COR- crude odds ratio, AOR- adjusted odds ratio, CI-confidence interval.* P- Value < 0.05, ** P- Value < 0.001, ^†^Coerced-transactional, forced, deception

Note: - variables not entered in the model because they were not found significant in bivariate analysis

In addition, compared to women who got married/cohabited at the age of 10-14 years, those who had at 15-19 years (AOR 3.41, 95% CI 1.31-8.89), 20-24 years (AOR 2.93, 95% CI 1.10-7.78), and ≥ 25 years were (AOR 4.26, 95% CI 1.27-14.2) more likely to report past 12 months experiences of IPVAW.

Furthermore, respondents were also asked whether their fore mothers were hit by fore fathers/husbands when they were children. Accordingly, witnessing inter-parental violence as a child were twice (AOR 2.00, 95% CI 1.54-2.56), and more than one and half times (AOR 1.66, 95% CI 1.17-2.37) more likely to report lifetime and current IPVAW, respectively. Also respondents whose husbands/partners themselves beaten by someone in their family during their childhood were nearly two times (AOR 1.89, 95% CI 1.17-3.03), and more than twice (AOR 2.11, 95% CI 1.41-3.15) as likely to report lifetime and current experiences of IPVAW (Table 5).

## Discussion

The objective of this study was to investigate the extent, patterns and associated factors of intimate partner violence against women. Most data on the prevalence of intimate partner violence comes from cross-sectional population based surveys [32]. Prevalence figures are liable to under or over reporting as the issue is surrounded by taboo and stigma [33,34].

The overall prevalence of IPVAW in this study is greater than any studies elsewhere [2,13,14], including studies from other parts of Ethiopia [17-21,35]. For instance, the findings from Butajira, Ethiopia, showed 71% and 54% of ever married/cohabited women experienced lifetime and current IPVAW. The study also showed that 49% and 29% of women had the experience of lifetime and past 12 months physical violence, respectively. In the same study, the corresponding figure for sexual violence was 59% and 44% [17]. These variations are likely due to the inclusion of psychological violence for measuring IPVAW into the present study. Cultural differences may also explain the discrepancy [22]. The prevalence of IPVAW in this study is also higher compared to the findings of Deribew from Agaro, Ethiopia used similar method reported the prevalence of IPVAW of 51.8% with 32%, 33%, and 46% for lifetime physical, sexual and psychological violence, respectively [18].

The high prevalence figures found for past-year and lifetime exposure for the three forms of violence indicate the fact that women's opportunities to end violence are few due to perpetration of violence being considered as normal male behavior. In other words, the subordinate role of women in the society and family allows violence to continue and keeps the divorce rates low especially among the low and middle income groups [36]. Moreover, the high prevalence in the present study might be due to the fact that women were interviewed by female data collectors who were known and familiar with the people in the community. This creates an opportunity for disclosure of violence by the women.

Another feature which was investigated in this study is the abundance of forced sexual acts in intimate relationships. It basically accounts for 58.7% during women's lifetime and 51.0% in the past 12 months which is far greater than 46% and 33% during lifetime and past 12 months in the study of Butajira, Ethiopia [17]. This showed a lot of non-consensual sex is happening in consensual marriage/cohabitation. On the other hand, the 2005 amended Criminal Code of Ethiopia [37] doesn't recognize forced sexual acts in marital relationship as crime. Accordingly it ignores the act of compelling a woman to submit to sexual intercourse within wedlock.

Despite various efforts which have been made by different governmental and non-governmental organizations to achieve MDG 3, much smaller variations were observed across the current, previous and lifetime experiences of IPVAW. However, much decline in the current practice has been expected.

The overlap of physical and psychological violence is the most commonly occurring form than the other two joint occurrences. This is consistent with studies from Nicaragua and South Africa [33,38] which is best explained as physical violence is often accompanied by psychological attacks, threatening and controlling behaviors [39]. Moreover, WHO multi-country study on VAW states that the most acts of physical violence reflect a pattern of abuse rather than an isolated incident [13]. Additionally, the present study shows the most severe violence seemed to be associated with greater overlapping of the different forms of psychological, physical and sexual violence that accounted for 56.9% of the total violence. This constitutes extremely serious situations and is much higher than 42% reported overlaps of physical and sexual violence in study from Butajira, Ethiopia [17].

There is no substantial overlap between psychological and sexual violence for women experienced any lifetime IPVAW. Again, of all abused women, 2.8% reported the experience of isolated sexual violence in their lifetime. This suggests that forced sexual acts alone by an intimate partner were not as prevalent in this population compared with isolated sexual violence by an intimate partner reported for other developing countries of WHO multi-county study on VAW that showed 31% in Butajira and 33% in Bangladesh [13]. The possible explanation is that women are less inclined to disclose sexual violence because it is shameful and very sensitive topic to be more pronounced in poor socio-economic country including Ethiopia.

Rural residents were less likely to report both lifetime and current experiences of IPVAW than urban residents. This is consistent with study from Philippines found a lower frequency of intimate partner violence among rural women [40]. This possibly indicated how women cope-up with urban life and what factors in the process of urbanization could be modified to decrease stress induced violence between women and men at intimate relationships. However, the finding of the current study is not consistent with the conclusion of WHO multi-country study and others that indicated rural localities presented higher rates than urban localities [13,41]. This might explain as gender relations in urban regions are more distant from traditional patterns and greater presence of women's movements and support services [42]. Similarly, rural communities are usually more conservative and the bedrock of the socio-cultural values of traditional societies that may promote the norms and tolerance of IPVAW. This is also true in the study area.

Older age of the respondents was significantly associated with increased risk of lifetime IPVAW corroborating other study [43]. This is possibly explained that the experiences of IPVAW are persistent from time to time in which the women report their cumulative experiences in lifetime. However, this is not consistent with Fernandez idea who described as the age of woman increases she often grows in social status as she becomes not only a wife, but also a mother and a socially influential member of her community. Thus, older women are less likely to report current experience of IPVAW than younger women [44].

The level of education for women and men were identified as statistically significant factor of IPVAW, which is consistent with studies elsewhere [13,42,45,46]. This is justified as educated women have greater range of choices in partners and able to negotiate greater autonomy and control of resources within the family. This in turn helps change norms and improves socio-economic conditions that capacitate them to protect themselves from IPVAW [42].

Compared to women who had no job, female headed respondents engaged into agricultural occupation and other activities appear to experience significantly lower levels of IPVAW. This could be explained that women are autonomous and empowered when they lead their livelihood while they are the head of the household. Indeed, this is not always true in Ethiopian context for which most of the households are headed by men.

This study also shows the increasing of the household socio economic status from poorest to richest is significantly associated with decreased risk of lifetime and current experiences of IPVAW while controlled for other variables. This association goes with Jewkes's explanation that poverty and associated stress are key contributors to intimate partner violence [42]. Here, poor socio-economic conditions contribute to violence in the family [47-49]. Although violence occurs in all socioeconomic groups, it is more frequent and severe in lower groups across such diverse settings of developed and developing nations [30,42].

Marriage by abduction increases the likelihood of experiencing lifetime IPVAW. This is so because abduction by itself is physically, psychologically, and sexually forcing a woman to have sexual intercourse often followed by marriage or cohabitation. This clearly indicates that male behaviors commonly associated with 'traditional' masculinity, which is strongly associated with IPVAW [50].

With this regard, for women who married/cohabited to polygamous husbands/partners, there is about four and two fold risks of experiencing lifetime and current IPVAW, respectively. This is consistent with the study findings from China and Uganda [51,52]. This could explain how IPVAW put women's reproductive health at risk. Hence, it was described for having multiple sexual partner could put women at increased risk for sexually transmitted infections together with violence contributes to psychological burden, low self esteem, feelings of embarrassment and humiliation [53].

On the other hand, husbands'/partners' extra marital affairs were more strongly associated with both lifetime and current experiences of IPVAW at bivariate level. Again, the associations of frequent use of alcohol by husbands/partners and increased risk of perpetrating their wives/partners is consistent with other studies elsewhere [47,54-56]. This could be attributed for heavy consumption of alcohol is thought to reduce inhibitions, cloud judgment, and impair ability to interpret social cues [57].

Moreover, male or female witnessing inter-parental violence during their childhood increases the risk of his/her later experience of IPVAW coincides with earlier findings from Ethiopia and other countries [17,49,55,58,59]. It has been suggested that witnessing inter-parental violence could lead to a normative understanding of violence and regarded as a fitting means of conflict resolution [42]. With this view, violence was confirmed as a learnt behavior that passes from generation to generation [60].

Similarly, men who witness parental violence are more likely to have attitudes that condone a husband's right to control his wife and to be violent to her [61]. This is to explain why a similar belief in male control of the family and the use of violence to achieve it exist in the world. It also explains why there are certain scenarios where intimate partner violence against women is seen as being justifiable by some men [62]. On the other hand, women who witness violence against their mothers (as children) are more likely to tolerate violence by their partners and respond in a passive manner. It is possible, therefore, that in the future these silent observers themselves will be victims or perpetrators of abuse and play a role in propagating IPVAW.

As to the limitation of this study, the cross-sectional nature could cause difficulty of determining the direction of the association between study variables. The associations could only be discussed in terms of plausibility. A further limitation is that the research team interviewed only women as proxy respondents for their husbands/partners, and hence relies on women's reports only. This can be biased when it comes to reporting on husbands'/partners' characteristic and the childhood experiences. However, the proxy respondents have been shown to produce reliable estimates in other contexts especially in asking husbands'/partners' behavior including frequency of alcoholic drinking [63]. Also, some husbands/partners might be conservative for telling their own childhood and current history to their partners.

As to the strengths of this study, it has got community-based nature and the respondents have been selected by random sampling technique with relatively large sample size. Again, the team already adopted standard and validated instrument of WHO multi-country study on VAW including special training of interviewers designed to maximize disclosure of violence across different social and cultural groups [64]. In addition, the team used interviewers and supervisors who have past experiences of data collection from their respective community. Because of all these measures, it was found an extremely high response and prevalence rate of the study.

To the best of the investigators' knowledge, this is the first to document and identify the patterns of IPVAW over the current, previous and lifetime experiences in Ethiopia. The information on pattern and severity of abuse might warrant the concerned body to guide the development of screening, treatment, and intervention programs for abused women and their perpetrators.

## Conclusions

Intimate partner violence against women is widely observed in the study area. Compared to similar studies the finding is among the highest. The study noted that more than three in four women were experienced at least one incident of IPVAW in their lifetime. Moreover, the patterns of IPVAW are similar across the time periods.

The joint occurrence of physical and psychological violence is the most commonly reported features of IPVAW. Moreover, overlapping of psychological, physical, and sexual violence accounted 56.9% of cases that indicate an extremely serious situation. Alarmingly, more than three quarter of women who experienced any physical violence had severe acts that could threaten them in their lifetime.

Area of residence, literacy status, socio-economic status, occupation, age of the respondents, and other cultural and behavioral factors were negatively or positively associated with IPVAW in the study area.

There is a need for protective efforts to break the norms that sustain women vulnerability in the society. Beside, the promotion of higher education and socio-economic development becomes vital. Additionally, education should target to shape children during their early age. This needs an urgent attention at all levels of societal organization including policymakers, stakeholders, professionals and other concerned body. Still interventions targeting behavioral and social factors promoting IPVAW should be instituted through the involvement of different stakeholders using a multi-sectoral approach and information dissemination tools. Moreover, extensive and longitudinal research is needed to validate the current findings.

## Competing interests

The authors declare that they have no competing interests.

## Authors' contributions

All three authors were responsible for the design and conduct of the study. The statistical analysis, the interpretation of findings and drafting of the manuscript were done by the three authors. The authors read and approved the final content of the manuscript.

## Pre-publication history

The pre-publication history for this paper can be accessed here:

http://www.biomedcentral.com/1471-2458/11/913/prepub
